# Resting‐State Hyper‐ and Hypo‐Connectivity in Early Schizophrenia: Which Tip of the Iceberg to Focus On?

**DOI:** 10.1002/brb3.71901

**Published:** 2026-11-13

**Authors:** David Tomecek, Marian Kolenic, Barbora Rehak Buckova, Jaroslav Tintěra, Filip Spaniel, Jiri Horacek, Jaroslav Hlinka

**Affiliations:** ^1^ National Institute of Mental Health Klecany Czech Republic; ^2^ Faculty of Electrical Engineering Czech Technical University Prague Czech Republic; ^3^ Institute of Computer Science of the Czech Academy of Sciences Prague Czech Republic; ^4^ Third Faculty of Medicine Charles University Prague Czech Republic; ^5^ Department of Medical Neuroscience Radboud University Medical Center Nijmegen the Netherlands; ^6^ Institute of Clinical and Experimental Medicine Prague Czech Republic; ^7^ Slovak Medical University Bratislava Slovak Republic

## Abstract

**Purpose:**

We examine brain connectivity alterations in early‐stage schizophrenia, with a focus on patterns of hyper‐ and hypoconnectivity. Although altered functional connectivity (FC) is well documented in schizophrenia, substantial inconsistencies remain regarding the location and direction of specific dysconnections. We hypothesize that early FC changes in schizophrenia include both decreases and increases and are correlated with symptoms and medication levels, with insufficient preprocessing potentially masking out some of these effects.

**Method:**

Using a large sample of 100 first‐episode schizophrenia patients (42 females) and 90 healthy controls (50 females), we compared FC across 90 brain regions defined by the Automated Anatomical Labeling atlas. We assessed medication effects and examined associations between FC changes and duration of untreated psychosis, duration of antipsychotic (AP) treatment, and symptom severity. In addition, we performed a systematic comparison of four functional magnetic resonance imaging data denoising strategies.

**Finding:**

Relative to controls, patients showed increased FC in 150 region pairs and decreased FC in 15 pairs, when using a “stringent” data denoising scheme. Despite this numerical imbalance, the overall distribution of FC changes remained balanced: Median FC was not systematically shifted, indicating no global bias toward hyper‐ or hypoconnectivity. Importantly, several FC alterations were significantly associated with symptom severity and AP medication.

**Conclusion:**

These findings indicate localized dysconnections embedded within a globally balanced connectivity profile in early schizophrenia. This balance was disrupted by less stringent denoising, leading to results dominated by hypoconnectivity, highlighting preprocessing as a major source of variability across studies.

## Graphical abstract

Stringent data denoising reveals a balanced distribution of hypo‐ and hyperconnectivity in schizophrenia (FES) that correlates significantly with clinical symptoms and antipsychotic medication. GSR forces balanced hypo‐ and hyperconnectivity but exhibits a weaker relationship between FC and clinical variables. Conversely, moderate denoising skews result toward hypoconnectivity, whereas the use of raw data introduces significant bias and obscures the relationship between connectivity and clinical variables.

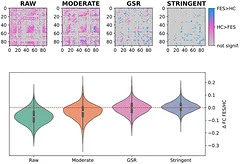

## 1 Introduction

Although a neurodevelopmental hypothesis for schizophrenia complemented by neurodegenerative processes following the onset of psychosis is now well established, it is not clear how these processes are linked to the brain dysfunction underlying specific schizophrenia psychopathology (Dauvermann et al. 2017). The converging evidence from brain imaging studies indicates that these processes lead to disordered brain inter‐regional functional connectivity (FC), and dysconnection represents the major candidate for the pathophysiological substrate of psychotic symptoms (Friston 2002). Concretely, the dysconnection concept suggests that the specific symptoms of schizophrenia can be described in terms of reduced or increased functional coupling between distinct brain regions (Friston and Frith 1995; Stephan et al. 2006). This concept is in line with earlier intuition referring to the title of the disease (i.e., “fragmented mind”), and it has been conceptualized in a neurobiological framework proposing a disruption of the anatomical and FC between brain areas as the neurobiological correlate of altered information processing (Friston and Frith 1995). To quantify such connectivity disruption, the concept of FC, formalized as temporal covariation of neural signals between spatially disparate brain areas (Friston et al. 1993), is often used.

The functional dysconnection in schizophrenia has been reported to affect connections of a multitude of brain regions, such as the frontal lobe, including language areas (Backasch et al. 2014; Benetti et al. 2009; Cole et al. 2011; Weinberger et al. 1994), sensory‐motor cortex (Kaufmann et al. 2015), temporal and limbic structures (Crossley et al. 2009; Meyer‐Lindenberg et al. 2005), and thalamus (Anticevic et al. 2014; Giraldo‐Chica and Woodward 2017; Guller et al. 2012; Woodward and Heckers 2016; Woodward et al. 2012). Despite the fact that neuroimaging studies strongly support the role of altered FC in schizophrenia, these reported findings are highly inconsistent; specific regions associated with disconnection in schizophrenia still remain controversial, and a robust conclusion has not yet been obtained. Some of the probable reasons for these inconclusive results are the heterogeneity of clinical samples regarding the stage of illness (T. Li, Wang, et al. 2016) and low sample sizes based on up to 40 participants on average (Dong et al. 2018). Indeed, a small sample size has been previously systematically documented to give rise to heterogeneous localized findings of white matter (WM) abnormalities in first‐episode psychosis (Melicher et al. 2015). We further conjecture that variability in the data preprocessing and analysis approaches might also contribute to the variability of the observed results (Burgess et al. 2016). The majority of previous resting‐state functional magnetic resonance imaging (rs‐fMRI) studies in schizophrenia reported hypoconnectivity between brain regions (Dong et al. 2018; S. Li et al. 2019), but hyperconnectivity has also been repeatedly observed in various disease stages (T. Li, Wang et al. 2016; Dong et al. 2018; González‐Vivas et al. 2019; Anticevic et al. 2015). Longitudinal studies of first‐episode schizophrenia (FES) subjects reported (partial) normalization in terms of increased fMRI activation after antipsychotic (AP) treatment (González‐Vivas et al. 2019). Another factor responsible for the heterogeneity of the findings is the methodological approaches focused on FC between a priori preselected regions, as contrasted with a whole‐brain analysis based on the parcellation according to suitable anatomical or functional brain atlases. Given these principal differences, the reported findings are strongly influenced by the a priori choice of the regions of interest (ROIs), which, in some cases, may not include all the pathophysiologically relevant areas. On the other hand, whole‐brain studies may suffer from the fact that the correction for multiple comparisons could bring false negative results, specifically in the case of underpowered studies. Our study aimed to remediate some of the above‐mentioned inconclusiveness and to utilize the whole‐brain FC analysis on a large cohort of 100 FES patients, ensuring high homogeneity in terms of chronicity and exposure to medication. The connectivity was calculated between all 90 brain regions comprising the Automated Anatomical Labeling (AAL) template image (Tzourio‐Mazoyer et al. 2002) and compared with a group of 90 healthy controls (HCs). On the basis of the previous evidence, we hypothesized that schizophrenia would show decreased connectivity between the candidate brain regions. Furthermore, we tested whether FC changes were moderated by the effect of medication and whether they were linked to clinical factors such as positive and negative symptoms of schizophrenia. In addition, we evaluated four different denoising strategies of the fMRI data, as this step potentially affects the resulting FC measures (Burgess et al. 2016).

## 2 Methods

### 2.1 Study Overview and Samples

In total, 190 subjects participated in the study: 100 FES patients (mean age = 28.78, standard deviation [SD] = 6.84, 42 females/58 males) and 90 healthy volunteers serving as controls (mean age = 27.88, SD = 6.83, 50 females/40 males). There were no significant differences between the patient and control samples in age and sex. In the patient group, at the time of the magnetic resonance imaging (MRI) scan, the average duration of untreated psychosis (DUP) was 3.23 months (SD = 4.79), and the average duration of AP treatment was 2.29 months (SD = 4.55). The study design was approved by the local Ethics Committee of the Institute of Clinical and Experimental Medicine and the Psychiatric Center Prague. All subjects provided written informed consent after receiving a complete description of the study.

The FES patients were diagnosed according to ICD‐10 criteria and structured MINI International Neuropsychiatric Interview (Sheehan et al. 1998). FES subjects were investigated during their first hospitalization in the Prague psychiatric hospitals with a catchment area of 1 million inhabitants. Patients were considered as FES if they fulfilled these criteria: (a) first hospitalization for schizophrenia, and (b) clinical interview identified first psychotic and/or prodromal symptoms of psychosis not earlier than 24 months ago (mean = 5.90 months, SD = 6.16).

The resting fMRI was performed at the initial stage of second‐generation AP therapy (mean 10 weeks of medication at the time of rs‐fMRI). The mean dose of chlorpromazine (CPZ) equivalents (Woods 2003) was 388.87 mg (SD = 235.44) per day. Psychometrics included the positive and negative symptom scale (PANSS) (Kay et al. 1987). Ninety HC subjects were recruited via a local advertisement; they had a similar socio‐demographic background as the FES to whom they were matched by age and sex.

The HC had a slightly higher number of years of education than the FES (HC: mean = 16.56, SD = 2.33; FES: mean = 13.51, SD = 2.46; *t* = 7.033, *p* < 0.001). HCs were evaluated with MINI (Sheehan et al. 1998) and were excluded if they had a lifetime history of any psychiatric disorder or a family history of psychotic disorders. Other exclusion criteria for both groups included a history of seizures or significant head trauma, mental retardation, a history of substance dependence, and any MRI contraindications. Written informed consent was obtained from all participants. See Table 1 for an overview of demographic and clinical characteristics.

**TABLE 1:** Demographic and clinical characteristics of the participants (mean/standard deviation [SD]).

	Age (years)	Sex (F, M)	Education (years)	DUP (months)	Dur. AP treat. (months)	Dur. sympt. (months)	CPZ (mg)
**HC**	27.88/6.83	50, 40	16.56/2.33	NA	NA	NA	NA
**FES**	28.78/6.84	42, 58	13.51/2.46	3.23/4.79	2.29/4.55	5.90/6.16	388.87/235.44

Abbreviations: AP, antipsychotic; CPZ, chlorpromazine; DUP, duration of untreated psychosis; FES, first‐episode schizophrenia; HC, healthy control.

### 2.2 fMRI Data Acquisition

Scanning was performed using a 3T Siemens Magnetom Trio MRI scanner located at the Institute of Clinical and Experimental Medicine in Prague, Czech Republic. Functional images were obtained using T2*‐weighted gradient echo‐planar imaging (GR‐EPI) sequence with blood oxygenation level‐dependent (BOLD) contrast with the following parameters: repetition time (TR) = 2000 ms, echo time (TE) = 30 ms, flip angle = 70°, voxel size = 3 × 3 × 3 mm, matrix size 48 × 64 voxels, 35 axial slices acquired continuously in sequential decreasing order covering the entire cerebrum, and 400 functional volumes in total. Total duration of the functional images acquisition was 13 min and 20 s. Moreover, for anatomical reference, a three‐dimensional T1‐weighted high‐resolution magnetization‐prepared rapid gradient echo (MPRAGE) image was acquired from each participant using the following parameters: repetition time (TR) = 2300 ms, echo time (TE) = 4.63 ms, flip angle = 10°, voxel size = 1 × 1 × 1 mm, matrix size 256 × 256 voxels, 224 sagittal slices covering the entire brain.

### 2.3 Data Preprocessing, Brain Parcellation, and FC Analysis

Functional MRI is a neuroimaging method that is based on measuring BOLD signal (Ulmer and Jansen 2020). One of the typical features of the fMRI data is the noise that is present in the raw BOLD signal (Biswal et al. 1995; Raichle 2015), which significantly limits the reliability of FC measures (Shirer et al. 2015). Typical artifacts, such as subject movements, arterial pulsation, respiration, and also the hardware of the MRI scanner itself, induce non‐neural temporal correlations in the BOLD (Pamilo et al. 2015), and relatively sophisticated data preprocessing is warranted to maximize the level to which the FC estimates reflect the underlying neuronal dynamics.

First, the structural and functional images were converted from DICOM to NIFTI format using the dcm2niix tool (X. Li, Morgan et al. 2016). The following preprocessing steps were performed with the functional data: functional realignment and unwarp; slice‐timing correction; outlier identification; direct segmentation and normalization; and functional smoothing—pipeline labeled “default preprocessing pipeline for volume‐based analyses (direct normalization to the Montreal Neurological Institute (MNI) space)” in CONN toolbox (Whitfield‐Gabrieli and Nieto‐Castanon 2012).

The denoising steps included the regression of six head‐motion parameters with their first‐order temporal derivatives and five principal components of WM and cerebrospinal fluid (CSF) signals using a component‐based noise correction method (CompCor) implemented in the CONN toolbox (Behzadi et al. 2007). Time series from defined ROIs were additionally linearly detrended and finally filtered by a band‐pass filter with cutoff frequencies 0.008–0.09 Hz. We shall refer to this preprocessing setup as the *stringent* denoising scheme. Our “stringent” denoising pipeline corresponds to the default denoising pipeline in the CONN toolbox, which incorporates the component‐based noise correction method (CompCor).

Given the continued debate regarding global signal regression (GSR) in fMRI preprocessing, we included it as an additional denoising scheme to assess the robustness of our findings to this commonly used preprocessing choice. For each participant, we generated a whole‐brain mask from anatomical segmentations to extract the average global signal. We then regressed out this signal along with six head‐motion parameters and their first‐order derivatives. Finally, the data were linearly detrended and band‐pass filtered with cutoff frequencies 0.008–0.09 Hz.

As an alternative denoising pipeline, closer to the practice in some studies (Argyelan et al. 2014; Wang et al. 2015), we used a *moderate* denoising scheme including six head‐motion parameters without their first‐order derivatives and only the mean time series of WM and CSF. This pipeline was performed without explicit linear detrending. However, time series were also finally filtered by a band‐pass filter with cutoff frequencies 0.008–0.09 Hz.

To isolate the impact of denoising, we conducted another analysis on the data that underwent the same default preprocessing pipeline in the CONN toolbox (Whitfield‐Gabrieli and Nieto‐Castanon 2012) as described above, while explicitly omitting all denoising procedures. This variant is referred to as the *raw*. The primary objective of this tiered selection was not to assess individual denoising methods in isolation but rather to illustrate how varying levels of preprocessing stringency—ranging from no denoising to standard‐practice stringent approaches—can fundamentally shift the results between hyper‐ and hypoconnectivity. In total, we evaluated four data denoising strategies: raw, moderate, stringent, and GSR.

### 2.4 Analysis

For each subject, to quantify the whole‐brain pattern of FC, we performed an ROI‐to‐ROI connectivity analysis by computing Pearson's correlation matrix among the regional mean time series extracted from 90 brain regions (excluding the cerebellar regions) of the AAL atlas (Tzourio‐Mazoyer et al. 2002) using the CONN toolbox (Whitfield‐Gabrieli and Nieto‐Castanon 2012). For all between‐group comparisons, we utilized the entire 90 × 90 ROI‐to‐ROI connectivity matrix, testing all pairwise connections. The resulting connectivity matrices were represented as Fisher's *z*‐transformed Pearson's correlation coefficients, calculated between the regional mean time series of each brain region. Prior work suggests that linear correlation is generally sufficient for capturing fMRI FC patterns (Hlinka et al. 2011; Raffaelli et al. 2024) relevant for clinical classification.

Although participants were balanced in terms of age and sex, these variables were further included as nuisance covariates in the linear regression model to further minimize their potential influence on FC.

To account for potential non‐normality in the distribution of FC values across subjects, non‐parametric statistical methods were employed. Accordingly, central tendency and dispersion are reported as median and median absolute deviation (MAD), respectively. We used a two‐sample Mann–Whitney *U*‐test to evaluate differences between groups. Spearman's correlation was used to determine the relation between the four subscales of the PANSS scale (Kay et al. 1987), AP medication at the time of the MRI scan (measured by CPZ equivalent), DUP, duration of AP treatment, and FC in patients. To increase statistical power and avoid severe multiple‐comparison corrections across all 4005 region pairs, analyses were restricted to ROI pairs demonstrating a significant between‐group effect. This two‐stage procedure avoids “double dipping” and controls Type I error rates because filtering on between‐group differences is mathematically independent of the subsequent within‐patient symptom severity tests (Bourgon et al. 2010). The resulting *p* values from each analysis were corrected for multiple testing by controlling the false discovery rate (FDR) (Benjamini and Hochberg 1995). Additional analyses with the moderate and raw denoising schemes were performed using the same analytical pipeline.

## 3 Results

### 3.1 The Difference Between HCs and Schizophrenia Patients

In the group comparison using the default, stringent denoising scheme, a difference was observed in a multitude of ROI pairs. A total of 150 ROI pairs had significantly greater FC (FDR‐corrected *p *< 0.05) in patients, whereas healthy volunteers exhibited significantly greater (FDR‐corrected *p* < 0.05) FC in 15 ROI pairs compared to patients (Figure 1). Despite the numerical prevalence of observed hyperconnections in patients, they did not show a difference in the median FC (median FC in patients = 0.0709, MAD = 0.0182, median FC in controls = 0.0653, MAD = 0.0209, *u* = 4007 [*z*‐score = −0.8663], *p* = 0.1932).

**FIGURE 1:**
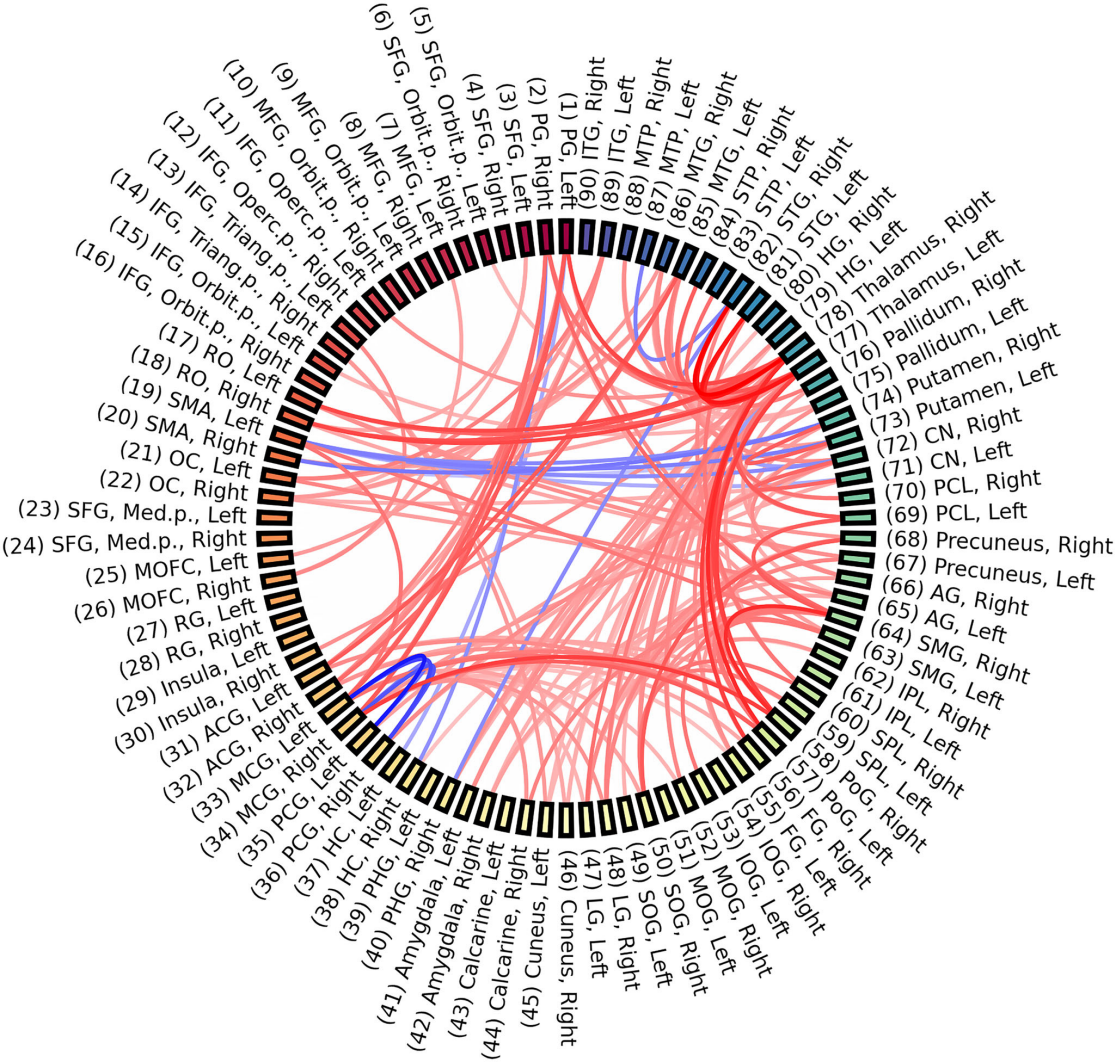
Difference between HC and FES; higher FC in healthy controls = blue, higher FC in patients = red, *p* < 0.05 (FDR‐corrected). HC, healthy control.

The most increased links in patients included a range of connections involving particularly the bilateral thalamus, right middle cingulate gyrus, right supramarginal gyrus, and left middle temporal gyrus (see Table S1 for the full list of affected connections). In the opposite direction, the decreased links in patients comprised especially the left supplementary motor area, bilateral posterior cingulate gyrus, right superior temporal gyrus, and left middle cingulate gyrus (see Table S2 for the full list of affected connections). See Figure 2 for a graphical overview of the group comparison of HCs and patients, including its stability with respect to additional control for inter‐subject variability in the amount of head motion; see Section 4 for details.

**FIGURE 2:**
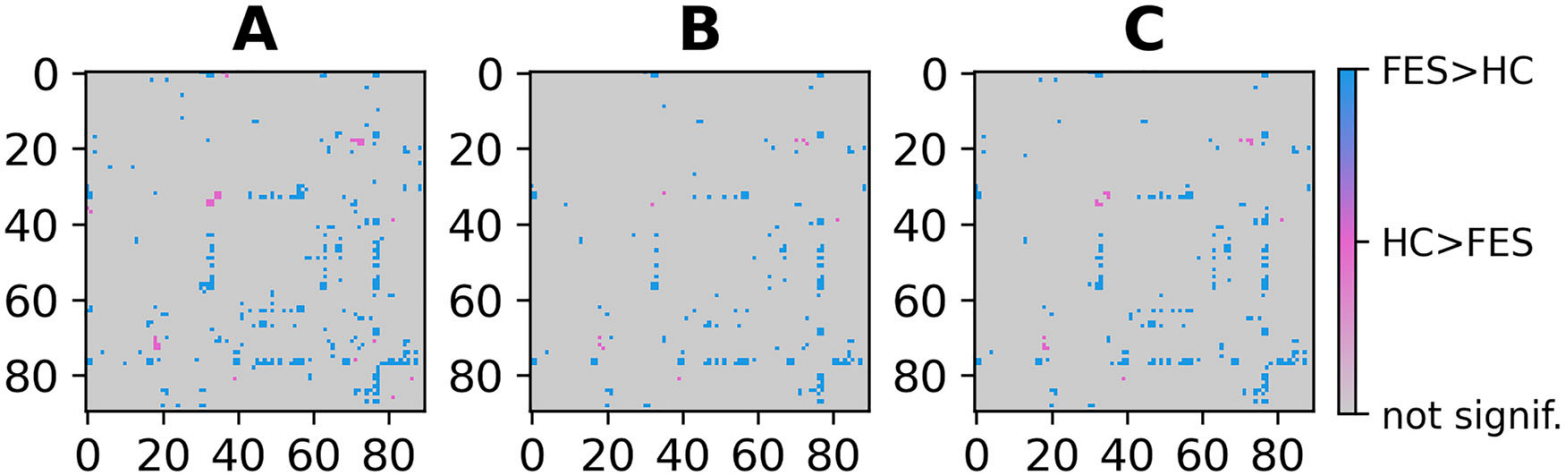
Difference between HC and FES. The significant differences after the *p* < 0.05 FDR correction are shown in blue (higher FC in patients) and pink (higher FC in healthy controls). (A) Default analysis; (B) analysis including additional inter‐subject correction for the amount of motion; (C) analysis including additional inter‐subject correction for the amount of motion, as well as rejecting outliers. See Section 4 for details of the additional analysis shown in the (B and C) panels. FES, first‐episode schizophrenia; HC, healthy control.

### 3.2 Association Between Symptom Severity, Medication, DUP, Duration of Antipsychotic Treatment, and FC

To increase the power by focusing on the most relevant candidate connections, we used a masking matrix of *p*‐values from a group comparison of healthy volunteers and patients to distinguish regions with significantly greater FC in patients.

We have observed a significant correlation between symptom severity measured by PANSS, AP medication, and FC; see Figure 3. PANSS positive scale significantly correlated with the FC between the right precentral gyrus and the right middle cingulate gyrus. PANSS negative scale significantly correlated with the FC between the right middle cingulate gyrus and the left lingual gyrus.

**FIGURE 3:**
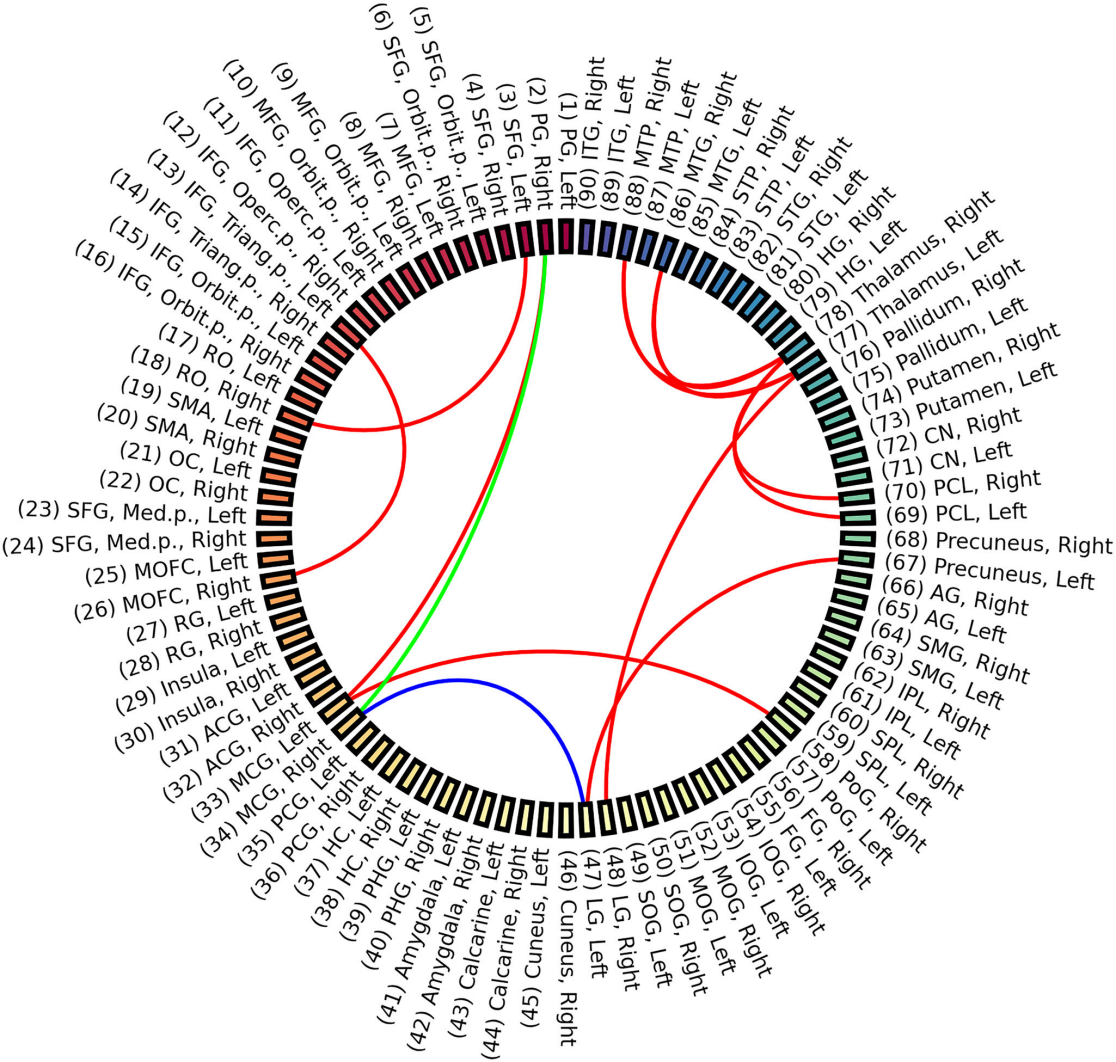
Association between antipsychotic medication, symptom severity, and functional connectivity. Antipsychotic medication significantly correlated with the functional connectivity of 12 ROI pairs (red links), PANSS positive scale significantly correlated with the functional connectivity of one ROI pair (green link), and PANSS negative scale significantly correlated with the functional connectivity of one ROI pair (blue link). Results with *p* < 0.05 (FDR‐corrected) are shown. HC, healthy control.

Further, we have observed a significant correlation between AP medication and FC. Regions with the most links included the bilateral thalamus, left middle cingulate gyrus, right middle temporal gyrus, and right middle temporal pole; see Table 2 for the full list of connections.

**TABLE 2:** Correlation between antipsychotic medication, positive and negative symptom scale (PANSS) positive, PANSS negative, and functional connectivity in patients; note that only *p* < 0.05 (false discovery rate [FDR]‐corrected) are reported.

Region 1	Region 2	Spearman *r*
***Antipsychotic medication***		
Thalamus, left	Middle temporal pole, right	0.33
Superior frontal gyrus, left	Rolandic operculum, right	0.32
Thalamus, right	Middle temporal gyrus, right	0.31
Thalamus, right	Middle temporal pole, right	0.31
Inferior frontal gyrus, triangular part, left	Medial orbital frontal cortex, right	0.31
Lingual gyrus, right	Thalamus, left	0.30
Lingual gyrus, left	Precuneus, left	0.30
Paracentral lobule, left	Thalamus, right	0.29
Precentral gyrus, right	Middle cingulate gyrus, left	0.29
Thalamus, left	Middle temporal gyrus, right	0.27
Middle cingulate gyrus, left	Postcentral gyrus, right	0.27
Paracentral lobule, right	Thalamus, right	0.27
***PANSS positive***		
Precentral gyrus, right	Middle cingulate gyrus, right	0.35
***PANSS negative***		
Middle cingulate gyrus, right	Lingual gyrus, left	0.33

We have also observed a significant correlation between AP medication and symptom severity measured by PANSS total (Spearman *r* = 0.33, *p* < 0.001).

Conversely, we found no relationship between the DUP, duration of AP treatment, and FC.

### 3.3 Effect of Data‐Denoising on Functional Connectivity

To evaluate the stability of the above‐presented findings with respect to the strictness of denoising, we also performed our analysis with raw and moderate denoising settings as well as with the GSR. In the denoising setting involving GSR, healthy volunteers exhibited significantly higher (*p* < 0.05 FDR‐corrected) FC in 258 ROI‐pairs compared to patients. Conversely, 167 ROI pairs had significantly higher (*p* < 0.05 FDR‐corrected) FC in patients; see Figure 5C. The most increased links in patients included bilateral middle cingulate gyrus, bilateral postcentral gyrus, and bilateral thalamus (see Table S9 for the full list of affected connections). In the opposite direction, the most decreased links in patients involved bilateral caudate nucleus, bilateral thalamus, and bilateral putamen (see Table S10 for the full list of affected connections).

The median connectivity difference between HCs and patients in the GSR denoising scheme was 0.0029 (median FC in patients = 0.0063, MAD = 0.0043, median FC in controls = 0.0092, MAD = 0.0042, *u* = 5510 [*z*‐score = −2.4253], *p* < 0.01).

In the case of GSR, we found a significant negative correlation with AP medication and FC between left superior occipital gyrus and left middle temporal pole.

In the moderate denoising, healthy volunteers exhibited significantly higher (*p* < 0.05 FDR‐corrected) FC in 1098 ROI‐pairs compared to patients. Conversely, 43 ROI pairs had significantly higher (*p* < 0.05 FDR‐corrected) FC in patients; see Figure 5B. The most increased links in patients included the bilateral thalamus, bilateral superior temporal gyrus, and left postcentral gyrus (see Table S3 for the full list of affected connections). In the opposite direction, the most decreased links in patients involved the right precentral gyrus, bilateral postcentral gyrus, left rolandic operculum, and the right paracentral lobule (see Table S4 for the full list of affected connections).

In stark contrast to the stringent denoising scheme, the median FC was greater in patients for the moderate denoising scheme. The median connectivity difference between HCs and patients in the moderate scheme was 0.0349 (median FC in patients = 0.1388, MAD = 0.0338, median FC in controls = 0.1737, MAD = 0.0477, *u* = 5762 [*z*‐score = −3.1351], *p* < 0.001). Indeed, the disease effect on FC indices was clearly biased toward hypoconnectivity in the moderately denoised dataset, whereas there was a relatively balanced hypoconnectivity and hyperconnectivity (with a slight prevalence of hyperconnectivity) observed when using the default stringent denoising scheme; see Figure 4D.

**FIGURE 4:**
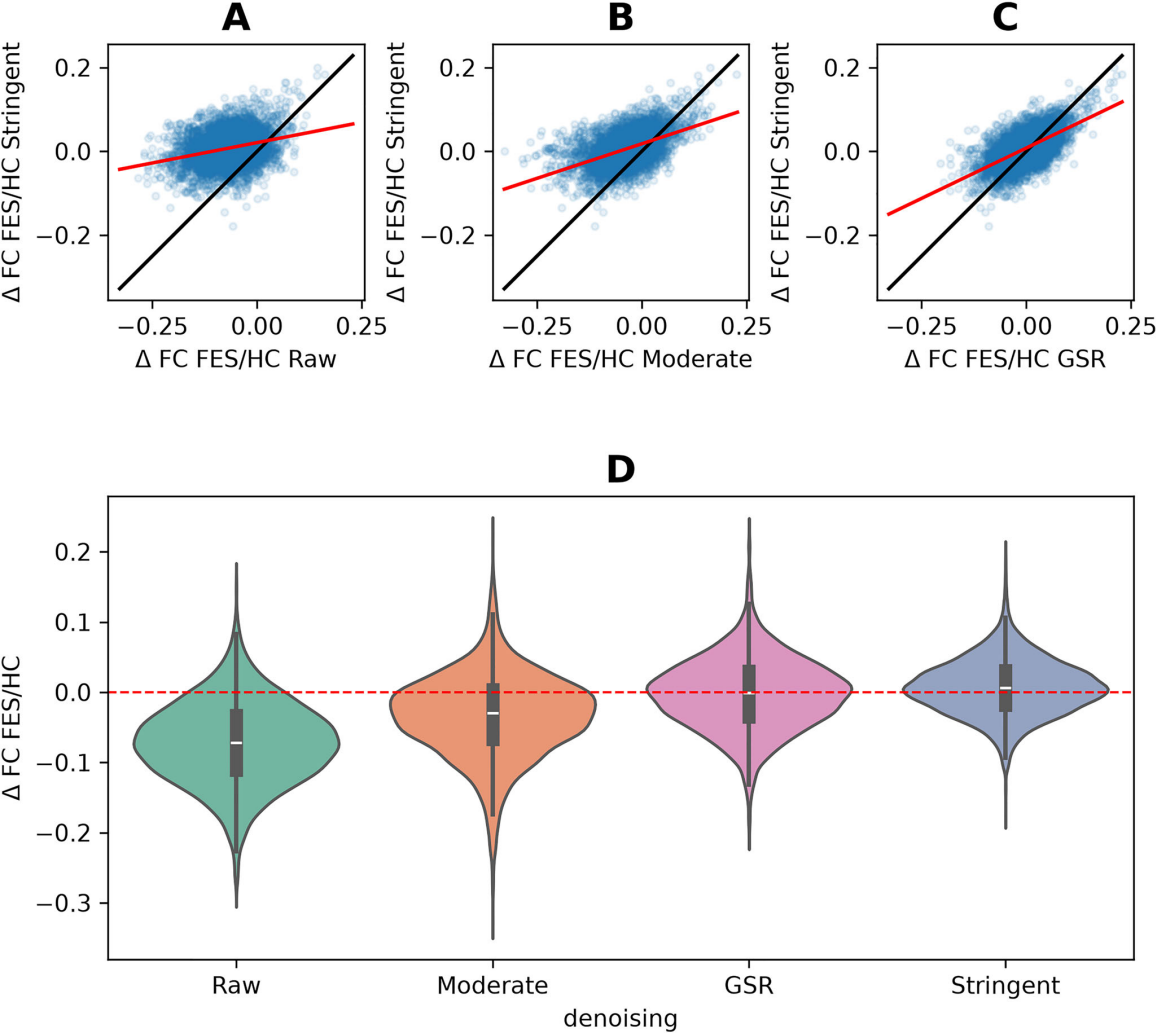
Comparison of the effect of disease on functional connectivity observed with the raw, moderate, GSR, and stringent denoising. (A) Scatterplot of the median FC difference between patients and healthy controls in the raw and stringent scheme. (B) Scatterplot of the median FC difference between patients and healthy controls in the moderate and stringent scheme. (C) Scatterplot of the median FC difference between patients and healthy controls in the GSR and stringent scheme. Black lines mark identity, and the red line corresponds to the best linear fit. Each dot corresponds to one pair of regions, showing the difference in their medians in the two denoising schemes. (D) Violin plot of the FC differences between healthy controls and patients in the raw, moderate, GSR, and stringent denoising schemes. FC, functional connectivity; FES, first‐episode schizophrenia; GSR, global signal regression; HC, healthy control.

In the case of moderate denoising, we found a significant correlation between FC and AP medication. Regions included the bilateral thalamus, bilateral rolandic operculum, and bilateral lingual gyrus (see Table S5 for the full list of affected connections).

In the raw denoising, healthy volunteers exhibited significantly higher (*p* < 0.05 FDR‐corrected) FC in 1281 ROI‐pairs compared to patients; see Table S6 for the full list of affected connections. Conversely, no ROI pairs had significantly higher (*p* < 0.05 FDR‐corrected) FC in patients; see Figure 5A. The median connectivity difference between HCs and patients in the raw scheme was 0.0611 (median FC in patients = 0.5025, MAD = 0.1132, median FC in controls = 0.5636, MAD = 0.1190, *u* = 5509 [*z*‐score = −2.4224], *p* < 0.01). The disease effect on FC indices was thus clearly biased toward hypoconnectivity in the raw scheme; see Figure 4D. We also observed no significant relation between FC and any of the clinical variables.

**FIGURE 5:**
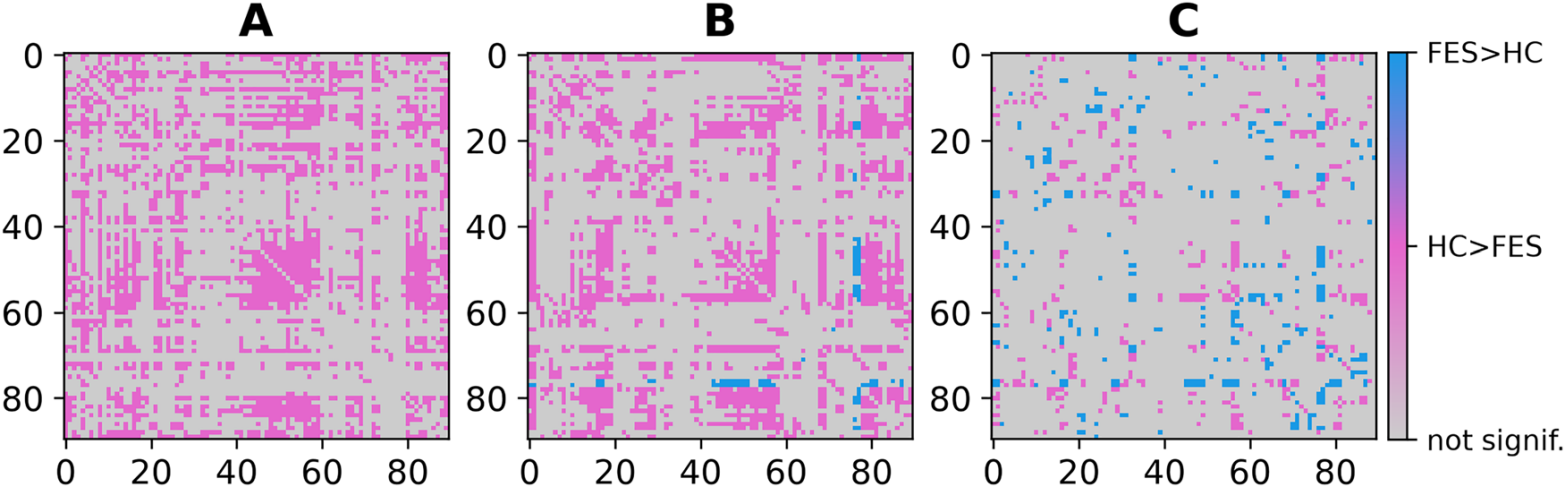
Difference between HC and FES for the (A) raw and (B) moderate (C) GSR denoising variants. The differences significant after the *p* < 0.05 FDR correction are shown in blue (higher FC in patients) and pink (higher FC in healthy controls). FES, first‐episode schizophrenia; HC, healthy control.

Despite these vast differences in the overall shift of FC between the two populations, the spatial localization of the disease effect observed with the four denoising schemes was strongly correlated (raw and stringent: Spearman *r* = 0.25, *p* < 0.001; moderate and stringent: Spearman *r* = 0.48, *p* < 0.001; GSR and stringent: Spearman *r* = 0.58, *p* < 0.001); see Figure 4A–C, respectively, for the scatterplot across region pairs.

### 3.4 Effect of Atlas Selection on FC

Although we initially utilized the AAL atlas to maintain comparability with a substantial body of established literature, we recognize the importance of validating these findings using more recent, functionally informed parcellations. To this end, we recomputed our primary analyses using both the Schaefer 100‐region atlas (Schaefer et al. 2018) and the Glasser atlas (Glasser et al. 2016). These supplementary between‐group analyses, see Table S7 for an overview, confirmed that our core findings were robust across alternative atlases (number of ROI pairs that showed significantly higher FC in HCs compared to patients): Schaefer 100‐Parcel atlas—stringent denoising resulted in 0/126 links, GSR 313/166 links, moderate denoising 1471/0 links, and raw denoising 1418/0 links; Glasser atlas—stringent denoising resulted in 3/96 links, GSR 1342/466, moderate denoising 13,043/0 links, and raw denoising 10,304/0 links. Moreover, using the Schaefer 100‐region or Glasser atlases yielded significant relationships between FC in patients and clinical variables only in combination with the stringent and GSR data denoising schemes; see Table S8 for more details.

## 4 Discussion

Using the stringent denoising scheme, we observed that individuals with FES showed a pattern of focal hyperconnectivity in thalamic, basal ganglia, and cingulo‐sensorimotor circuits, combined with cortical hypoconnectivity. This profile aligns with models of schizophrenia that conceptualize early psychosis as a disturbance of hierarchical cortico–subcortical integration, with thalamic and striatal systems exhibiting increased (possibly compensatory) reactivity against a background of impaired cortical coordination (Anticevic et al. 2015; Wengler et al. 2020). This hybrid pattern replicates prior work documenting thalamo–sensorimotor hyperconnectivity in schizophrenia (Anticevic et al. 2015; Woodward et al. 2012) and suggests an early imbalance in hierarchical predictive processing.

The most increased links in patients involved the bilateral thalamus, globus pallidus, bilateral middle cingulate gyrus, bilateral supramarginal gyrus, right superior occipital gyrus, and bilateral precuneus. These findings mirror the “thalamocortical dysconnectivity” framework proposed by Anticevic et al. (2014) and the results of meta‐analyses reporting increased thalamic coupling to sensorimotor cortices in psychosis. According to these models, thalamic hyperconnectivity may reflect insufficient top‐down regulation from prefrontal and hippocampal systems, leading subcortical hubs to over‐signal or over‐synchronize with cortical targets. Notably, a subset of the hyperconnected edges identified here was also implicated in symptom severity and medication effects, suggesting that these subcortical–cingular–motor circuits may represent a core network phenotype of early schizophrenia.

In contrast, the most decreased links in patients involved circuits linking the supplementary motor area and precentral cortex with the hippocampus, caudate, and putamen. This pattern is consistent with meta‐analytic evidence for widespread hypoconnectivity across fronto‐parietal, hippocampal, and cortico‐striatal networks in schizophrenia (Dong et al. 2018; S. Li et al. 2019). These networks support predictive processing, contextual integration of prior information, and precision‐weighted gating of sensory signals (Friston 2005; Corlett et al. 2019). Their reduced functional integration in FES aligns with computational theories proposing that weakened cortical priors and impaired hippocampal‐prefrontal coordination degrade the system's ability to maintain stable internal models and suppress noisy bottom‐up prediction errors (Sterzer et al. 2018; Sigurdsson and Duvarci 2016). In this context, thalamic and striatal hyperconnectivity may arise as maladaptive attempts to stabilize cortical dynamics in the face of insufficient cortico‐hippocampal control, a view that is compatible with theoretical work showing that hierarchical heterogeneity of cortical microcircuits shapes large‐scale neural dynamics and stability (Demirtaş et al. 2019). Our findings also align with longitudinal evidence in AP‐naive FES showing widespread baseline dysconnectivity and that AP exposure in AP‐naive FES is associated with increased FC primarily between the thalamus and the rest of the brain (Chopra et al. 2021). A striking and unifying observation across all analyses was the role of the mid‐cingulate cortex (MCC). MCC appeared as a major hyperconnected hub in patients, but also as the key node associated with both PANSS symptom dimensions and CPZ‐equivalent AP exposure (see below). This convergence suggests MCC as the principal clinically relevant region in our dataset. The MCC is an integrator of salience, motor preparation, effort allocation, and executive control, functions that are highly disrupted in psychosis. Its involvement here strongly supports theories emphasizing abnormalities in salience signaling and motor relevance attribution in schizophrenia (Shackman et al. 2011; Hoffstaedter et al. 2014; Kapur 2003). This is further supported by studies demonstrating aberrant recruitment of the medial cingulate/anterior cingulate cortex (ACC) region during cognitive control and motor‐preparatory tasks in psychosis (Heckers et al. 2004).

Furthermore, brain hyperconnectivity correlated with both symptom severity and AP medication in subjects at their first episode of psychosis. The positive symptom dimension (PANSS positive) was associated with FC between right precentral gyrus and right MCC, indicating enhanced coupling between motor cortex and a core salience/motor‐control hub (Shackman et al. 2011; Du et al. 2019; Kapur 2003). This pattern is highly compatible with Kapur's aberrant salience model and with contemporary models linking psychosis to dysregulated interactions between salience networks and motor/output systems. Conversely, the negative symptom dimension (PANSS negative) was associated with FC between the right MCC and the left lingual gyrus in our sample, suggesting altered coupling between a cingulate salience/control hub and visual–perceptual cortex. Although this specific MCC–lingual link is not a canonical finding, it is consistent with broader evidence that negative symptom dimensions relate to salience‐network dysfunction and abnormal salience–default mode (and salience–sensory) communication in schizophrenia (Hare et al. 2019; Palaniyappan and Liddle 2012). These findings are consistent with the literature suggesting that negative symptom dimensions are associated with cingulate/salience‐network dysfunction and with altered coupling between salience/control systems and sensory–perceptual networks, potentially contributing to impaired contextual appraisal.

This pattern most likely reflects a combination of illness severity, given that CPZ‐equivalent dose is clinically titrated and therefore is often confounded with positive symptom severity in naturalistic samples (Troisi et al. 1997; Ringen et al. 2019), and medication‐driven modulation of salience‐related motor circuitry, in line with evidence showing AP effects on thalamo‐cortical and cingulate networks (Chopra et al. 2021; Sarpal et al. 2015). Importantly, Chopra et al. demonstrated that DMN–limbic normalization can occur even without medication, whereas drug exposure specifically alters thalamo‐cingulate circuits. Our findings extend this by showing that in a naturalistic FES sample, AP exposure was most strongly associated with the same motor–salience connections that also tracked positive symptom severity in our data. The overlap between CPZ‐related and PANSS Positive‐related connectivity further supports the interpretation that apparent medication effects may partly reflect underlying symptom burden.

A further limitation is that putative pharmacoresistance was not systematically followed; although the first‐episode, early‐course design reduces the likelihood of established treatment resistance, emerging non‐response may have contributed to clinical heterogeneity.

Our findings may also be interpreted within the framework of corollary discharge and impaired sense of agency, potentially linking altered precentral–MCC connectivity associated with positive symptoms to disturbances in monitoring self‐generated actions (Frith 2000). More broadly, the cingulo‐opercular network has recently been reconceptualized as an action‐mode network supporting the initiation and maintenance of goal‐directed behavior (Dosenbach et al. 2025), with previous evidence of its dysconnectivity in schizophrenia (Tu et al. 2012). Within this framework, our distinct MCC associations with positive and negative symptoms may reflect different aspects of disrupted action‐related processing, including agency and, more tentatively, goal‐directed behavior. However, these mechanistic interpretations remain hypothetical, as resting‐state connectivity cannot directly assess corollary discharge or agency.

It is worth mentioning some technical considerations of the present study. Several denoising strategies were discussed in recent papers. Although a unique consensus has not been reached in the field, the implementation of a more stringent denoising scheme has become a standard practice (Power et al. 2014; Patel et al. 2014), motivating our default choice. To quantify FC, in line with common practice, we use Pearson's linear correlation coefficient, as it was shown to be sufficient under standard conditions, as with the current dataset (Hlinka et al. 2011; Raffaelli et al. 2024). A possibly fruitful further direction would indeed lie in extension to mapping the consequences or analogies of our findings to other network characteristics previously shown to be altered in clinical high‐risk and early‐illness schizophrenia individuals, in particular to dynamic FC (Mennigen et al. 2019) and amplitude of low‐frequency fluctuations or graph‐theoretical functional dysconnectivity characterization (Tang et al. 2019). On the other side, utilizing the full FC matrix has shown an advantage in machine learning classification of schizophrenia above and beyond some advanced methods such as topological data analysis (Caputi et al. 2021).

There was a considerable difference between the stringent, GSR, moderate, and raw denoising schemes in the number of significant ROI pairs in each group comparison. Both raw and moderate denoising schemes resulted in a higher number of ROI pairs that showed significantly higher FC in HCs compared to patients (1281/0 in raw and 1098/43 in moderate), with GSR exhibiting more balanced results (258/167), whereas in the stringent denoising scheme, the proportion changed as we observed a slightly higher number of ROI pairs exhibiting hyperconnectivity in patients (15/150). Note that the use of a substantially stricter denoising scheme than used in a range of studies in the literature may explain some of the deviation of our results with respect to the field. Despite the differences between denoising strategies, median connectivity matrices are strongly correlated within each group (the correlation of FC between raw and stringent was 0.5301 (*p* < 0.001) in healthy individuals and 0.5681 (*p* < 0.001) in patients, between moderate and stringent was 0.8798 (*p* < 0.001) in healthy individuals and 0.9181 (*p* < 0.001) in patients, and between GSR and stringent was 0.9219 (*p* < 0.001) in healthy and 0.9021 (*p* < 0.001) in patients). Moreover, the difference between the groups, that is, the disease effect, is strongly correlated (across region pairs) between the denoising strategies (raw and stringent: Spearman *r* = 0.25, *p* < 0.001; moderate and stringent: Spearman *r* = 0.48, *p* < 0.001; GSR and stringent: Spearman *r* = 0.58, *p* < 0.001); see Figure 4A–C, respectively, for the scatterplot across region pairs. Although we found overlapping ROI‐pairs between the stringent and moderate denoising that exhibited correlation with AP medication (viz., regions involving the bilateral thalamus, right middle temporal pole, right middle temporal gyrus, and right lingual gyrus), we found no relationship between FC and any of the selected clinical variables when the raw data were used, confirming that minimal standard preprocessing is needed to reduce noise in the data. Although GSR resulted in a numerically more balanced distribution of hyper‐ and hypoconnectivity (an effect not surprising given its natural well‐known tendency to create balance of positive and negative FC already at individual level), it appears it compromised the sensitivity of the analysis to clinical associations. In our sample, the stringent scheme captured an association with PANSS scores and AP medication that was largely obscured when the global signal was regressed, indicating that the global mean may contain biologically relevant information.

To ascertain that the results were not driven by any residual artifacts due to differences in head motion, we also carried out a control analysis where mean framewise displacement (FD), the aggregated measure of subject motion, was included as a covariate of no interest. The results again remained qualitatively equivalent, suggesting that the effect of motion on FC is relatively small compared to the effect of disease; see Figure 2B. The results remained largely unchanged after the additional removal of the 14 subjects who showed maximum head motion larger than 1.5× interquartile range (IQR), the Tukey criterion for detection of outliers (Tukey 1977); see Figure 2C. The difference in the number of ROI pairs with significantly changed FC could be due to lower degrees of freedom in the additional corrected analyses.

Although our study captures a cross‐sectional “snapshot” of FC at the onset of psychosis, we recognize that schizophrenia is a progressive disorder, and these connectivity patterns likely evolve over time. Our current data do not allow for a direct assessment of how the localized balance of hyper‐ and hypoconnectivity observed in this first‐episode sample might transition into the more widespread hypoconnectivity often reported in chronic populations. Although a formal longitudinal analysis is beyond the scope of the current work, investigating the temporal dynamics of these circuits remains a critical objective for our future research, which will aim to determine how these early‐stage alterations track with disease progression and chronic symptom development.

## 5 Conclusions

Our study using a whole‐brain FC approach on a relatively large sample has shown a relatively balanced, yet spatially resolved, picture of hyperconnectivity and hypoconnectivity in first‐episode psychosis while demonstrating a significant association between the affected connection strengths and clinical symptoms and cumulative dosage of AP medication. Our results support a model in which early schizophrenia combines cortical hypoconnectivity with subcortical and cingulo‐sensorimotor hyperconnectivity, forming a hybrid dysconnectivity profile that is closely tied to symptoms and treatment. Importantly, we have shown that this balanced picture gets substantially skewed in the direction of dominantly observed hypo‐ or dysconnectivity when applying more moderate data‐denoising schemes, whereas the relation to symptoms is rendered insignificant. Although the results of all approaches are in overall qualitative agreement, including with localization of most commonly reported disease connectivity effects in the literature, taking into account the results obtained with the current stringent denoising may provide a stronger relation to clinical variables and contribute to the understanding of the diversity of previously reported results, as well as the interpretation of schizophrenia as a dysconnection disease in general.

## Author Contributions

**Filip Spaniel**, **Jiri Horacek**, and **Jaroslav Hlinka** are responsible for conceptualization. **David Tomecek** and Jaroslav Hlinka are responsible for methodology. David Tomecek is responsible for software. David Tomecek and Jaroslav Hlinka performed the validation. David Tomecek, Jiri Horacek, and Jaroslav Hlinka are responsible for the formal analysis. David Tomecek, **Marian Kolenic**, **Barbora Rehak Buckova**, Filip Spaniel, Jiri Horacek, and Jaroslav Hlinka are responsible for investigation. **Jaroslav Tintěra**, Filip Spaniel, and Jaroslav Hlinka provided the resources. David Tomecek, Jaroslav Tintěra, Filip Spaniel, Jiri Horacek, and Jaroslav Hlinka are responsible for data curation. David Tomecek, Marian Kolenic, Barbora Rehak Buckova, Jiri Horacek, and Jaroslav Hlinka wrote the original draft. David Tomecek, Marian Kolenic, Barbora Rehak Buckova, Jaroslav Tintěra, Filip Spaniel, Jiri Horacek, and Jaroslav Hlinka reviewed and edited the manuscript. David Tomecek is responsible for the visualization. Jaroslav Tintěra, Filip Spaniel, Jiri Horacek, and Jaroslav Hlinka performed the supervision. Jaroslav Tintěra, Filip Spaniel, and Jaroslav Hlinka administered the project. Filip Spaniel, Jiri Horacek, and Jaroslav Hlinka are responsible for funding acquisition.

## Funding

The publication was supported by ERDF‐Project Brain Dynamics, No. CZ.02.01.01/00/22_008/000464, Czech Health Research Council Project No. NU21‐08‐00432, and by the Ministry of Health, Education and Sports of the Czech Republic program Cooperatio 038, Neuroscience, Charles University.

## Disclosure

This manuscript has been posted on a preprint server, BioRxiv, under doi: https://doi.org/10.1101/2024.09.20.613853.

## Ethics Statement

The study was originally approved by the Ethics Committee of the Prague Psychiatric Center in 2011 (No. 69/11) and subsequently continued under the Ethics Committee of its successor institution, the National Institute of Mental Health, with repeated protocol amendments. The procedures and data analyzed in the present study were included in the protocol from the outset.

## Conflicts of Interest

The authors declare no conflicts of interest.

## Supporting information




**Supplementary Table**: brb371901‐sup‐0001‐S1.xlsx

**Supplementary Table**: brb371901‐sup‐0002‐S12.xlsx

**Supplementary Table**: brb371901‐sup‐0003‐S13.xlsx

**Supplementary Table**: brb371901‐sup‐0004‐S14.xlsx

**Supplementary Table**: brb371901‐sup‐0005‐S15.xlsx

**Supplementary Table**: brb371901‐sup‐0006‐S16.xlsx

**Supplementary Table**: brb371901‐sup‐0007‐S17.xlsx

**Supplementary Table**: brb371901‐sup‐0008‐S18.xlsx

**Supplementary Table**: brb371901‐sup‐0009‐S19.xlsx

**Supplementary Table**: brb371901‐sup‐00010‐S10.xlsx

## Data Availability

The data that support the findings of this study are available from the corresponding author upon reasonable request.
